# Toward a new conceptualization of resilience at work as a meta-construct?

**DOI:** 10.3389/fpsyg.2023.1211538

**Published:** 2023-09-14

**Authors:** Anaïs Galy, Denis Chênevert, Evelyne Fouquereau, Patrick Groulx

**Affiliations:** ^1^Pôle Santé HEC Montréal, Department of Human Resources, HEC Montréal, Montréal, QC, Canada; ^2^QualiPsy EE 1901, Psychology Department, University of Tours, Tours, France

**Keywords:** work, resilience, emergence, resource, multilevel, theoretical article

## Abstract

Organizations of all kinds are faced with multiple demands for adaptation of increasing frequency and amplitude due to such factors as reorganizations, climate change, pandemics, and labor shortages. This new reality requires our organizations to anticipate, adjust, and demonstrate resilience. The study of resilience at work relies on the comprehension of how organizational systems, as well as their work collectives and members, manage to overcome adversity without suffering from irreversible damage. However, the study of this phenomenon of interest contains grey areas concerning both its definition, its conceptualization, and the dynamic processes that underlie it. This theoretical paper addresses these different issues by providing first, a conceptual content analysis of the most frequently used definitions and second, a new conceptualization of resilience at work as a resource, either individual or collective. Moreover, we suggest a multilevel, dynamic, and virtuous conceptual approach to resilience at work, relying on both bottom-up and top–down flows. Accordingly, we formulate different theoretical propositions upon which future empirical research can draw to analyze the relationships between individual, team, and organizational resilience. Building on a conservation of resources lens, we offer a novel contribution to the resilience in the workplace literature, by providing an integrative and multilevel theory of resilience at work that highlights both the processual and interpersonal nature of its emergence, and the organizational levers that can foster it.

## 1. Introduction

As complex sociotechnical systems embedded in a disruptive global context, organizations are increasingly exposed to highly unstable external environments. Confronted with change, disruption, turbulence, and even crisis, organizations must fight for their survival, which largely depends on the adjustment capacity of the actors and groups who compose them (Sutcliffe and Vogus, 2003; Vogus and Sutcliffe, 2007). Therefore, resilience at work appears to be a key resource for dealing with a wide variety of adversarial situations that require organizational adaptation, either in isolated instances or recurrently. Whatever the nature of these events, they stress the need to mobilize workers as well as work collectives to face the inherent challenges.

Beyond the apparent usefulness and interest of the construct of resilience at work as well as the growing interest it has aroused in various academic circles, there are still numerous disagreements about its definition, conceptualization, and consequently its measurement (Linnenluecke, 2015; Khan et al., 2019; Hartwig et al., 2020; Stoverink et al., 2020; Hartmann et al., 2020a,b). At the center of the latest academic debates, there are central questions about the nature of resilience at work as a capacity and as a process, about its genesis and its development over time, and even about its transfer from individual to collective levels — that is, from individual persons to teams, and organizations (Stoverink et al., 2020; Raetze et al., 2021, 2022). The lack of a collective understanding on resilience has hindered both empirical and theoretical advancements, thereby impeding the implementation of effective organizational measures to enhance positive adversity management at all levels within organizations.

Therefore, this article aims to answer the call from numerous authors (e.g., Linnenluecke, 2015; Khan et al., 2019; Hartwig et al., 2020; Stoverink et al., 2020; Hartmann et al., 2020a,b; Raetze et al., 2021, 2022) for theoretical and conceptual clarification on resilience at work. Its simple yet comprehensive conceptualization provides an integrative view of resilience at work and the processes involved, from a positive psychology perspective (Luthans et al., 2007). More precisely, we propose a definition beyond the one that has been used in research before now. Indeed, we offer an important conceptual clarification that addresses the existing fragmentation of knowledge on this concept. Recognizing the importance of comprehending the intrinsic nature of resilience and the factors contributing to its development, this study offers a second theoretical contribution by presenting an original and integrative theoretical model. Notably, it fills a gap in the existing research by integrating all three levels of resilience—individual, team, and organizational—into one dynamic meta-construct. By considering both bottom-up and top–down mechanisms, this article highlights the significance of time in the emergence and growth of resilience.

## 2. Definitions and conceptual clarification of resilience at work

Rooted in the physical sciences, the concept of resilience was initially introduced by researchers whose goal was to describe the capacity of a material to return to its initial state after being exposed to a force or an impact. During the transposition of the concept to the social sciences, in disciplines like psychology and management, this initial core principle has been preserved. Indeed, many studies characterize resilience as a mechanism like a spring that allows individuals or groups to overcome an adverse event and then return to their initial state (Hartwig et al., 2020; Stoverink et al., 2020; Hartmann et al., 2020a,b). In this article our focus is resilience at work; therefore, our theorization applies to organizations, teams, and work collectives, as well as to individuals acting as the cornerstones of these sociotechnical systems.

According to Walker and Avant (2005), a conceptual analysis should always be informed by an attentive examination of the various definitions of the given concept. Therefore, we scrutinized some of the most popular definitions of resilience (see Table 1) and used the following methodology to select and analyze their content. To identify these 22 definitions, we first searched for the most recent literature reviews and conceptual articles published both in top journals and academic handbooks. All of these selected references are marked by a star in the bibliography. Second, we manually extracted the definitions of resilience presented in all these publications. Third, we followed a complementary approach by conducting an upward and downward search across the selected articles. For the upward process, we identified the key articles cited, screened them, and extracted the definitions. For the downward process, we searched, screened, and extracted content from sources citing the primary references that were identified. All definitions were then listed in a table and duplicates were deleted prior to the content and frequency analysis.

**Table 1:** Review of key definitions of resilience.

Authors (Year)	Level	Definitions	Key concepts
Fletcher and Sarkar (2013)	At work	As the ability of employees to positively manage and overcome negative events at work.	Individual ability, negative event
Wildavsky (1988)	General	As learning from adversity how to do better (p. 2), and the “capacity to cope with unanticipated dangers after they have become manifest, learning to bounce back” (p. 77).	Capacity, learning, coping, bounce back
Weick et al. (1999)	General	As the ability to ‘bounce back’ (p. 14).	Ability, bounce back
Luthar et al. (2000)	General	As a dynamic process encompassing positive adaptation within the context of significant adversity.	Dynamic process, positive adaptation, adversity
Weick and Sutcliffe (2001)	General	As the ability to bounce back (p. 14).	Ability, bounce back
Sutcliffe and Vogus (2003)	General	As the maintenance of positive adjustment under challenging conditions.	Positive adjustment
Sutcliffe and Vogus (2003)	General	As a characteristic or capacity of individuals or organizations, or more specifically (a) the ability to absorb strain and preserve (or improve) functioning despite the presence of adversity (both internal adversity —such as rapid change, lousy leadership, performance and production pressures — and external adversity, such as increasing competition and demands from stakeholders).	Characteristic, capacity, positive adjustment, adversity
Sutcliffe and Vogus (2003)	General	As an ability to recover or bounce back from untoward events.	Ability, bounce back
Britt et al. (2016)	General	As a positive adaptation, through which the entity returns to a steady state of well-being or performance or even bounces beyond it.	Positive adaptation, bounce beyond
Williams et al. (2017)	General	Used to describe organizations, systems, or individuals that are able to react to and recover from duress or disturbances with minimal effects on stability and functioning.	Ability, react, recover, disturbance, minimal consequences
Williams et al. (2017)	General	As the process by which an actor (i.e., individual, organization, or community) builds and uses its capability endowments to interact with the environment in a way that positively adjusts and maintains functioning prior to, during, and following adversity.	Capability, process, positive adjustment
Hartmann et al. (2020a,b)	General	As a dynamic process encompassing positive adaptation within the context of significant adversity (p. 918).	Dynamic process, positive adaptation, adversity
Luthar et al. (2000)	Individual	As a dynamic process allowing positive adaptation in the face of major adversity (p. 543).	Dynamic process
Meyer (1982)	Organization	As an organization’s ability (embodied in the existence of resources, ideologies, routines, and structures) to absorb a discrete environmental jolt and restore prior order.	Ability, absorb jolt, restore prior order
Vogus and Sutcliffe (2007)	Organization	As the maintenance of positive adjustment under challenging conditions such that the organization emerges from those conditions strengthened and more resourceful.	Positive adjustment, bounce beyond
Barasa et al. (2018)	Organization	As involving both adaptation and transformation of the organizational system undergoing a crisis. These authors also emphasize the critical role of organizational resilience, in that it allows systems to survive and adapt to increasingly unstable environments.	Adaptation, transformation, overcoming crisis
Khan et al. (2019)	Organization	As an organization’s ability to absorb strain and preserve or improve functioning, despite the presence of adversity.	Ability, strain, adversity
West et al. (2009)	Team	As the team’s ‘capacity to bounce back from failure, setbacks, conflicts, or any other threat to well-being that they may experience’ (p. 253).	Capacity, bounce back
Morgan et al. (2013)	Team	As a dynamic process of a psychosocial nature that protects a group of individuals from the potentially negative effects of collectively encountered stressors. It incorporates processes by which team members use their individual and collective resources to adapt positively when faced with adversity (p. 552).	Dynamic process, positive adaptation
Flint-Taylor and Cooper (2017)	Team	As the processes of managing pressure effectively across the team as a whole [. ..], that further strengthen the capacity of the team to deal with future challenges in adversity (p. 130).	Process, managing pressure, capacity, adversity
Gucciardi et al. (2018)	Team	As an emergent outcome characterized by the trajectory of a team’s functioning, following adversity exposure, as one that is largely unaffected or returns to normal levels after some degree of deterioration in functioning.	Emergent outcome, adversity, bounce back
Stoverink et al. (2020)	Team	As a team’s capacity to bounce back from adversity-induced process loss.	Collective capacity, bounce back, adversity

Building on this approach, we identified three key components which constitute the main axes of the content analysis grid that we have developed. First, all of these definitions are based on a specific interpretation of the nature of the phenomenon. Second, each author indicates in which situations this construct applies by specifying the nature of the disruptive events which provoke its application. Third, these definitions always indicate the expected consequences or outcomes when groups or individuals display resilience. To highlight the main points of divergence, we conducted a textual and thematic content analysis of the 22 definitions presented in Table 2. The detailed content analysis also emphasizes the frequency in occurrence of the diverse subthemes identified within each of the three main components. The analysis of these distinctions is important because it clarifies the conceptual divergence observed in the literature and in the associated academic debates that ensue (Linnenluecke, 2015).

**Table 2:** Frequency analysis of the main components of the definitions.

	Number of occurrences	Frequency (%)
Nature of the phenomenon		
Ability, Capacity, Capability	**15**	68
Dynamic process	**5**	23
Characteristic	1	5
Positive adaptation or adjustment	**4**	18
Emergent outcome	1	5
Nature of the situation		
Adversity	**11**	50
Challenging conditions	**3**	14
Negative Events	1	5
Unanticipated dangers	1	5
Strain / stressors	**2**	9
Untoward events	1	5
Duress or disturbances	1	5
Environmental jolt	1	5
Crisis	1	5
Failure	1	5
Setbacks	1	5
Conflicts	1	5
Threats	1	5
Pressure	1	5
Process loss	1	5
Function of the construct		
Positively manage or overcome negative events	1	5
Bounce back, restore prior order	**10**	45
Positive adaptation / adjustment	**7**	32
Absorb strain, manage pressure	2	9
Preserve, maintain functioning	2	9
Bounce beyond, emerge more resourceful, improve or strengthen functioning.	**4**	18
React and recover	1	5
Minimal effect on stability and functioning	1	5
Survive	1	5

*N* = 22 definitions. Please note that a definition can have several occurrences for each component analyzed. For each component of the definitions, the most salient elements from the frequency analysis are highlighted in green. The darker the green, the more frequently the element is used.

The divergences in the nature of the phenomenon center around three main subthemes and suggest a somewhat contrasting conceptualization of resilience: as a capacity, as a dynamic process, or as a positive adaptation. As for the nature of the disruptive event, it is mostly characterized by the term “adversity,” which can be defined as “a state or instance of serious or continued difficulty or misfortune.”^1^ As for the other situational streams, they mainly indicate that this key component is a difficulty or even a stressor. Finally, regarding the expected outcomes associated with the mobilization of resilience, the weighting of the different perspectives seems more balanced. Thus, a significant proportion of the definitions allude to the notion of bouncing back, although there are some definitions that emphasize the notion of achieving a positive adaptation and others that even stress the notion of bouncing beyond difficulties or setbacks. This last notion is generally defined as the ability to emerge from adversity stronger or with more resources (Vogus and Sutcliffe, 2007; Britt et al., 2016).

Given these considerations, it seems essential to unify and clarify the definition and conceptualization of resilience at work as well as the three levels at which it occurs, namely the individual, the collective, and the organizational levels. This conceptual fragmentation has led to major problems with respect to both theoretical and empirical generativity, two fundamental aspects of research (Suddaby, 2010; Podsakoff et al., 2016). Moreover, it is essential to ensure that both the definitions and the conceptualization of the construct are consistent with the theoretical models on which they rely. Thus, by applying all of the aforementioned recommendations to this theorization, we conceptualize the construct in the following way. First, this study is anchored in the Conservation of Resources theory of Hobfoll (1989, 2001), and it relies on a processual vision of resource management mechanisms. Accordingly, we conceptualize resilience as a resource, or even an aggregation of resources, specifically when referring to it at the collective levels of conceptualization. Second, as the disruptive situation is the key antecedent to resilience (Raetze et al., 2022), we believe that considering its high prevalence in the resilience literature, as highlighted in Table 2, the term adversity represents a corner stone on which most of the authors agree. As a rare element regarding this often qualified as scattered research domain (Linnenluecke, 2015), we thus suggest and build on this terminology to refer to these situational demands. Third, as we explain in depth in the rest of this article, we conceptualize resilience as a developmental construct relying on the bouncing-beyond perspective. Accordingly, our position does not align with the bounce-back conceptualization that has been used by numerous authors over the last 20 years. Since a parsimonious definition should refer to the key elements of the concept while limiting itself to the core characteristics, we retain the notion of positive adjustment to refer to the mechanisms and the outcomes that relate to resilience.

In line with this conceptualization, we define resilience at work as a pool of positive resources — both individual and collective — that allow organizational systems and their actors to positively adapt while facing adversity. Moreover, there is a need to distinguish effective resilience, which relates to a past and proven ability to overcome a given adversarial situation, from prospective resilience, which pertains to a projection of the potential ability of individuals or groups to positively adapt when confronted with adversity (Moenkemeyer et al., 2012; Todt et al., 2018). This distinction is crucial as numerous authors have called for theoretical models that integrate the chronological components of resilience (e.g., Gucciardi et al., 2018; Khan et al., 2019; Hartwig et al., 2020; Stoverink et al., 2020). To our knowledge, Duchek (2020) is one of the rare researchers who presents an integrative view of the temporal issues associated with resilience in the workplace. This seems essential because it emphasizes the importance of both the situational and the contextual external factors in the genesis of resilience, while also highlighting the active process undertaking by the involved entities. Indeed, just because individuals or teams have shown resilience when confronted with a specific past event does not necessarily imply that resilience will manifest in their present or future management of aversity.

Furthermore, as stated by Raetze et al. (2022), “Resilience is just one concept to explain how different organizational entities overcome adversity” (p. 881). Hence, when conducting a conceptual analysis, it is necessary to distinguish the construct under scrutiny from overlapping others to ensure conceptual clarity (Walker and Avant, 2005). Accordingly, in the following sections, we contrast resilience with well-established related constructs, namely adaptation and learning. According to many scholars, adaptation is probably the concept that shares the most commonalities with resilience (Raetze et al., 2022). However, if adaptation implies changes in cognitions, attitudes, or behaviors, it can lead to very distinct outcomes, such as positive or negative adaptation. When facing an environmental stimulus, positive adaptation is generally a synonym of an improvement in individual or collective efficiency while managing an event. However, on the opposite side of the continuum, negative adaptation generally results in a disequilibrium between the demands of the environment and the attitudinal and the behavioral dispositions deployed by individuals or collectives. Hence, resilience refers to a positive adaptation to an adverse event, whereas avoidance (Roth and Cohen, 1986) or resistance (Oreg, 2006) are both manifestations of a negative adaptation. Yet, resilience at work differs from positive adaptation, for it corresponds to events of a much higher magnitude than the ordinary and recurrent demands encountered in daily work life.

When examining the conceptual overlap between resilience and learning, it becomes evident that both concepts share common processes. Experiencing plays a crucial role in both learning and resilience, where individuals can utilize past knowledge to take action in the learning process, and those exposed to adversity learn from their successes and failures in the resilience process. Despite these similarities, resilience and learning diverge in their contexts and outcomes. Resilience revolves around facing and surpassing adversity, while learning focuses on acquiring knowledge in specific domains. The result of learning is the acquisition of new knowledge, whereas resilience’s outcome is the capacity to rebound and transcend adversity. Consequently, employees may acquire new knowledge through learning but struggle to apply it in practical situations, while they may adapt to adversity without necessarily gaining new knowledge. Moreover, learning remains an ongoing and cyclical process, while resilience tends to emerge episodically, predominantly in response to adversities.

### 2.1. Individual resilience

Even though research focusing on individual resilience did not begin recently, there is still no consensus with regard to its definition, conceptualization, and measurement (Hartmann et al., 2020a,b). Some authors suggest considering individual resilience as a trait — something relatively stable and over which neither individuals nor organizations have any room for maneuvering (Hartwig et al., 2020; Hartmann et al., 2020a,b) — whereas other researchers posit that resilience is a capacity that can be enhanced (Fletcher and Sarkar, 2013), or even that it is a dynamic process (Luthar et al., 2000). According to this last conceptualization, Luthar et al. (2000) refer to resilience as “a dynamic process encompassing positive adaptation within the context of significant adversity” (p. 543). Our perspective on resilience aligns with this latter definition, viewing this construct as a dynamic process. It is grounded in the fact that all organizational stakeholders can actively contribute to their self-resilience by developing attitudes and behaviors that enhance both its emergence and deployment (for detailed reviews, see Hartmann et al., 2020a,b; Raetze et al., 2022).

We define individual resilience at work as a positive individual resource that enables an employee to positively adjust, in both attitudinal and behavioral terms, when confronted with adversity. Not only does this resource reflect the past adversarial events that the individual has successfully overcome, but also it relies on the potential for positive adjustment in managing future turbulence. Thus, employees’ resilience is an effective potential that can be mobilized if needed and that reflects the exposure to previous adverse events. This highlights the roles of time and experience in the genesis and deployment of resilience, when conceptualized as a developmental construct. Accordingly, individual resilience can be compared with a muscle increasingly developing over the course of training. Employees build an enhanced potential for positive adaptation that relies on their experience with adverse situations and thus depends on both the number and the intensity of the encountered adverse event(s). In occupational settings, this is also reflected in the development of knowledge and various skills, which highlights the aforementioned conceptual bond linking resilience and learning.

Yet, most studies relating to individual resilience at work describe resilience as a rebound capacity and not as a developmental construct (Hartmann et al., 2020a,b). These researchers (e.g., Stephens et al., 2013; Stoverink et al., 2020) suggest that when faced with an adverse situation, employees tend to experience a disequilibrium and then return to their initial state of balance once successful adaptation has been achieved. As mentioned by Richardson (2002), this vision is closely related to the concept of homeostasis (Cannon, 1939) — initially developed in physiology — which refers to a general state of equilibrium, or base level, that each individual perpetually seeks to regain when confronted with either internal or external demands that cause a state of imbalance. However, we contend that this idea cannot apply to resilience, since its core principle relies on a pool of positive resources that emerge from the variety of encountered adversarial events as well as the associated acquired knowledge. According to our conceptualization of resilience as a dynamic construct, a resilient worker can never return to their base level after facing a crisis, because this initial state no longer exists. Indeed, as we describe in the second section of this article, resilience at work is a developmental construct that emerges over time (see Proposition 1).

### 2.2. Team resilience

The topic of team or collective resilience at work is much more recent in this stream of research (Hartwig et al., 2020; Hartmann et al., 2020a,b; Raetze et al., 2022). As with individual resilience, team resilience is characterized by various conceptual divergences. Hartmann et al. (2020a,b) even mention that conceptual developments concerning this stream of research are still in their infancy and that no consensus exists for now. Nevertheless, the existence of a large number of recent literature reviews on the topic of team resilience highlights the willingness of authors to clarify and unify the conceptualization of this construct (Chapman et al., 2020; Hartwig et al., 2020; Hartmann et al., 2020a,b). Along these lines, Gucciardi et al. (2018) and Stoverink et al. (2020) have proposed integrative conceptual models that are aimed at summarizing the key antecedents and outcomes of team resilience.

The definitions and the conceptualization of team resilience are numerous and heterogeneous (Hartwig et al., 2020). Some authors conceptualize team resilience as an emerging dimension that is rooted in employees’ resilience and that progressively transforms into a shared capacity of the members of a given team (West et al., 2009; Gucciardi et al., 2018). Based on this approach, team resilience relies largely on the individual resilience of the various members composing the team. Nevertheless, this vision is far from consensual among researchers, thus leading various authors to propose a somewhat different approach to team resilience, namely conceptualizing it as an emergent construct that relies both on internal and external characteristics. According to this conceptual stream, team resilience is not only a function of the individual resilience of each team member, but also a function of the team’s characteristics, its dynamic processes, and team members’ specific behaviors (Marks et al., 2001). The definition proposed by Morgan et al. (2013) synthesizes this perspective as follows: “a dynamic psychosocial process which protects a group of individuals from the potential negative effect of stressors they collectively encounter. It comprises processes whereby team members use their individual and collective resources to positively adapt when experiencing adversity” (p. 552).

We define team resilience as a positive collective and shared resource that enables one or several groups of employees to positively adjust — in both attitudinal and behavioral terms — when confronted with adversity. Hence, we do not conceptualize team resilience as a simple agglomeration or even the sum of the resilience of individuals that comprise the team. Rather, the idea is to conceptualize individual resilience as a first-order phenomenon and team resilience as a second-order phenomenon in an integrative view of the emergence of resilience at work, which is grounded in the understanding that resilience at work is a metaconstruct existing at different levels of abstraction. In agreement with Stoverink et al. (2020), we believe that the key distinction between individual and team resilience concerns the degree of complexity and interdependence required for the genesis of collective resilience. Therefore, as suggested by Hartmann et al. (2020a,b) as well as Gucciardi et al. (2018), we conceptualize team resilience as an emergent process that arises from interactions among team members, but results in a higher-level manifestation, as compared with individual resilience. Furthermore, we adopt a multi-deterministic conception of team resilience, which includes the individual characteristics of team members (e.g., individual resilience), the intensity and quality of interpersonal relationships within the team (e.g., cohesion), the level and quality of perceived support (e.g., organizational support, managerial support, peer support), and the nature of the context.

### 2.3. Organizational resilience

At a higher level of analysis, we consider how the organization, as an entity composed of proactive actors, will succeed in overcoming disturbances or crises that occur in its internal or external environment. In this line of thinking, Barasa et al. (2018) highlight the critical role of organizational resilience, as it allows systems to survive and adapt to increasingly volatile environments. This last level of analysis of the metaconstruct referring to resilience at work is described by Vogus and Sutcliffe (2007) as “the maintenance of positive adjustment under challenging conditions such that the organization emerges from those conditions strengthened and more resourceful” (p. 3,418).

We define organizational resilience as a positive organizational resource, which is shared by organizational actors, and which enables the organizational system to positively adjust when it is confronted with adversity. We emphasize the developmental nature of organizational resilience — that is, we assume that as the organizational system undergoes adversity, it learns from its successes and failures, thus increasing both its resilience state and its resilience potential (Duchek, 2020). Along these lines, Barasa et al. (2018) suggest that this concept involves both the adaptation and the transformation of the organizational system undergoing a crisis. Thus, through its past adverse experiences, the organizational system evolves, learns, and capitalizes on new resources that can in turn contribute to more effective adaptation to future adverse situation.

Even though organizational resilience shares common characteristics with team resilience, such as its function and its interactional principles, it is distinct from the latter, notably due to a lower level of interdependence among organizational actors at the organizational level (Britt and Sawhney, 2020; Stoverink et al., 2020). The two most notable differences relate to (1) the fact that structurally, the organizational level involves a relational network whose members are often less tightly knitted, and (2) the idea that decision-making processes do not involve the same issues for a team as they do for an organization (Stoverink et al., 2020). At the organizational level, the ability to mobilize workers and teams to overcome an adverse situation is closely related to the ability of decision-makers to instill messages that encourage the necessary mobilization of individual and collective resources as part of the common aim of getting through the crisis (Barasa et al., 2018).

Additionally, numerous literature reviews and much theorization regarding organizational resilience emphasize its material and structural components, for example the effects of the strategic planning capacities, the governance system in place, the HRM practices, or even the financial resources available to the organization (Lengnick-Hall et al., 2011; Barasa et al., 2018; Khan et al., 2019; Vakilzadeh and Haase, 2021). Conversely, some authors (Hobfoll et al., 2018; Khan et al., 2019) suggest that human capital is fundamental to the genesis of workplace resilience. Our conceptualization is aligned with this latter characterization. Indeed, our focus is grounded in the understanding that the human and social dimensions are considered cornerstones in the process of building a resilient organizational system. Consequently, in acknowledgement of the interdependence of the diverse and numerous organizational actors, we emphasize the relational nature of the processes involved (Barton and Sutcliffe, 2023). This is supported by Khan et al. (2019), who suggest that organizational resilience is a mechanism grounded in social processes and occurring between the various stakeholders of the organization (i.e., ingroup and outgroup). In contrast with a global or even unitary perspective of organizational resilience (Barasa et al., 2018; Khan et al, 2019) highlight the extent to which the resilience of an organization is intensively dependent on the resilience of its components, such as the different teams or the departments. As such, these authors posit the existence of a relationship between team and organizational resilience. This perspective aligns with our own in that we suggest that the three levels of resilience (i.e., individual, team, organizational) are intrinsically related processes. Accordingly, as discussed in the second part of this article, we conceptualize each level of resilience as a necessary but not sufficient condition for the emergence of higher-level resilience (i.e., team, organization); this is due to the emergent nature of this phenomenon.

Given the conceptualization and the different definitions of workplace resilience that we have presented in this first section, we now examine the emergent nature of this phenomenon, which is considered a key resource for all organizational systems.

## 3. A dynamic and emergent process

Organizational systems represent complex socio-technical environments characterized by the interdependence of actors, work collectives, and existing structures. Consequently, it is important to investigate the ways in which various factors express and influence each other according to the prism through which they are studied, something Kozlowski and Klein (2000) highlight in the following way: “Neither single-level perspective can adequately account for organizational behavior” (p. 7). Indeed, individual (i.e., micro) entities are embedded in both organizational (i.e., macro) contexts and interpersonal (i.e., meso) dynamics. This is reflected by Hox et al. (2017), when they point out that organizations are “hierarchical system of individuals nested within groups, with individuals and groups defined at distinct levels of that hierarchical system” (p. 1). As such, multilevel analyses attempt to account for the complexity as well as the interdependence of members and structures within workplaces (Kozlowski and Klein, 2000). Doing so enables the understanding of the dynamic components of these systems, specifically by proposing the study of ascending, descending, or even temporal (i.e., longitudinal) effects.

### 3.1. Theoretical underpinnings of resilience emergence

Bottom-up processes describe how lower-level properties interact and then emerge to form higher-level collective phenomena (Kozlowski and Klein, 2000). With these, the purpose is to understand how individual resilience contributes to the emergence of both team resilience and organizational resilience, while also considering the links between team resilience and organizational resilience.

To characterize emergent processes, Kozlowski and Klein (2000) propose a typology of emergence that relies on two types of processes — composition and compilation — considered as two opposite poles of a continuum of emergence. In brief, composition is based on the principle of isomorphism in the emergence of a higher-level phenomenon. Conversely, compilation is based on the principle of discontinuity in the translation of a construct to a higher level. Nevertheless, as emphasized by these authors, many emergent phenomena are ultimately grounded in the hybridization of these two mechanisms and not in the perfect expression of them in their purest forms.

These principles appear to be particularly salient in the study of resilience at work, as they allow for an integrative understanding of both the commonalities and dissimilarities of resilience across the different levels of analysis in organizational settings. Hence, as mentioned by Britt and Sawhney (2020), although they are presented as three distinct constructs, organizational, team and individual resilience exhibit a certain level of homotheticity in the mechanisms deployed (e.g., adversity detection, sense-making). However, while presenting a certain level of isomorphism across its different levels, resilience does not simply express as a pure translation (i.e., referent shift; Chan, 1998) when it emerges at higher levels of abstraction. Indeed, the different levels of resilience are functionally equivalent (i.e., they serve common purposes) but at different levels of the organization. Yet, team resilience, like organizational resilience, also presents collective aspects that are absent from the conceptualization at the individual level (e.g., interdependence, work climate).

Thus, the emergence of this construct at collective levels originates from the heterogeneity of the profiles, the expertise, and the experiences of the members of the organization, all through a unique combination of individual and collective resources. This is what Gucciardi et al. (2018), speaking of the emergence of team resilience, summarize as follows: “This form of emergence is analogous to a puzzle, where the individual pieces represent unique human capital resources of individual members that fit together to generate an overall picture that makes sense” (p. 735). This indicates that we are faced with an emergent phenomenon based on compilation mechanisms. However, the emergence of resilience is also reflected in the existence of commonalities, which carry over into the translation of the construct at higher levels of abstraction, as outlined by Gucciardi et al. (2018): “With regard to composition emergence, team resilience is the function of both individual-level interpretations of the team’s capability to resist, bounce back, or recover from deteriorations in functioning following adversity, and the shared perception of these interpretations among team members” (p. 755). Indeed, the emergence of collective resilience (e.g., team, organization) also relies on composition mechanisms. Therefore, it is through the interactions and shared experience among employees that the convergence of perceptions occurs over time, thus allowing the adaptive potential of the group and their resilience to emerge from a collective perspective at the team level and at the organizational level (Kozlowski and Klein, 2000; Gucciardi et al., 2018).

In summary, the emergence of resilience at work, both at the team and at the organizational levels, reflects a complex phenomenon at the intersection of compilation and composition processes that Bliese (2000) describes as a “fuzzy composition process” (p. 369). If the multilevel theoretical perspective highlights the nature of the phenomenon at different levels, thus answering the *what* questions, it does not explain the mechanisms by which this dynamic translation operates, which would answer the *how* questions.

Indeed, conceptualizing resilience at work as a meta-construct existing at three different organizational levels also involves the need to clarify how these levels interact and the underlying mechanisms that explain their emergence at these different levels. To this end, we draw on the Conservation of Resources theory (COR) proposed by Hobfoll (1989, 2001) as well as some more recent theoretical developments relating to the potential for resource crossover among individuals and groups at work (Westman, 2001; Chen et al., 2015; Hobfoll et al., 2018). Accordingly, we conceptualize the emergence of resilience as a bottom-up process wherein resilience evolves from a first-level individual construct to a second-level shared construct (i.e., team level) and then finally to a third level (i.e., organizational level).

### 3.2. Conservation of resources theory

The COR theory (Hobfoll, 1989, 2001) postulates that the individual actively seeks to shape their environment to obtain maximum pleasure and success. It is grounded in a positive conception of the individual, who is given significant power to act, guided by their motivation to accumulate, protect, and maintain resources, whether personal, social, or material. In this theory, the conceptualization of a resource is broad, which allows it to be applied to a variety of contexts and levels of analysis (Hobfoll et al., 2018). Applying the core principles of this theory to our theoretical development, we posit that workplace resilience is a key resource for organizational systems and all their members. In addition, the COR theory highlights the existence of resource agglomeration, called resource caravan, which suggests that resources do not exist individually but instead tend to regroup for both individuals and organizations (Hobfoll, 2011b). As defined by Hobfoll (1989, 2001), when a resource is gained or lost, it brings with it the resources attached to it, which leads, respectively, to the emergence of a positive or a negative spiral. Resource caravans serve as bridges (i.e., passageways) that promote the creation and dissemination of resources throughout all the levels of the organizational system (Hobfoll et al., 2018).

#### 3.2.1. COR theory and resilience emergence

Since 2011, many publications building on the COR theory (e.g., Westman, 2001; Hobfoll, 2011b; Chen et al., 2015; Hobfoll et al., 2018) have focused on incorporating resilience as a key contributor to understanding the different mechanisms linking various resources (i.e., crossover). However, most of these studies consider resilience as the consequence of conservation and development of resources. Here, we adopt a different analytical lens by conceptualizing resilience as a resource rather than a consequence. It is coherent with the conception of a resource proposed by Hobfoll et al. (2018), which states that a resource should support individual or collective goal attainment. This conceptualization is relevant, as it allows for an analysis focusing on resilience and aiming at identifying the underlying mechanisms that explain not only its genesis but also its diffusion across all levels of the organizational system. Moreover, due to the polysemous nature of the concept of resilience at work as well as its anchoring in a wide range of heterogeneous first-level phenomena, we contend that resilience can be viewed as an emerging meta-resource (Duchek, 2020).

#### 3.2.2. Individual resilience emergence

Regarding individual resilience, the core principles of the COR theory apply as follows. First, in accordance with Chen et al. (2015), we distinguish between two distinct components of resilience: the psychological and the behavioral. However, our conceptualization is somewhat different from that of these researchers, as we suggest that the psychological components relate to the individual’s belief in their capacity or their potential to overcome adversity and that the behavioral components reflect the transposition of this belief into active or even proactive actions that allow the individual to face the adversarial situation. It is through the combination of these two components that an employee can positively adjust when confronted with a difficult situation. Accordingly, a resilient worker is a worker who is able to mobilize the relevant resources at their disposal to face adversity, such as a professional crisis (Britt et al., 2016; Plimmer et al., 2022). Therefore, this mechanism refers to the mobilization of positive resources, which in turn leads to the emergence of a new positive resource once the obstacle has been overcome: the employee’s individual resilience (Vogus and Sutcliffe, 2007). This is consistent with the core principles of the COR theory, by highlighting the deployment of gain spiral for individuals. Indeed, the emergence of individual resilience at a given point in time operates as a facilitator for the management of future crises, creating a bridge toward the future that in turn facilitates the maintenance and even development of existing individual resources (Sutcliffe and Vogus, 2003; Hobfoll et al., 2018; Stoverink et al., 2020; Bardoel and Drago, 2021). Thus, these successes enable the employee to develop a pool of positive resources, which will then facilitate their management of the potentially threatening events (Sutcliffe and Vogus, 2003). Consequently, we formulate the following proposition:

*Proposition 1*: Workers’ individual resilience is a developmental construct capitalizing on previous overcome adversity. Thus, there is a positive relationship between a worker’s resilience at time t and their level of individual resilience at time *t* + 1.

#### 3.2.3. Team resilience emergence

According to these same principles, individual resilience acts as a lever in the genesis and the maintenance of team resilience. This individual resource is a facilitator that promotes the emergence of relational and contextual conditions that foster the genesis of team resilience (Barton and Sutcliffe, 2023). From this perspective, employees’ resilience paves the way for the sharing of experiences, of resources, or even of emotions among team members (Westman, 2001; Chen et al., 2015; Hobfoll et al., 2018).

Inter-individual interactions facilitate the emergence of a shared understanding of the reality and a collective belief in the group’s capacity. These elements then serve as a foundation upon which the potential and effective resilience can be built (Hartmann et al., 2020a,b). The sharing of these psychological and behavioral components, which exist both through contagion effects and imitation processes (Stoverink et al., 2020), leads to a form of collective effervescence, which allows for the creation of a shared pool of resources among team members (Stephens et al., 2013; Meneghel et al., 2016). This is what Chen et al. (2015) describe as the commerce of resources, which refers to both material and immaterial exchanges of products and resources among the members of a group and which can result in the exchanging of ideas, opinions, or even feelings. Therefore, it is through these mechanisms of voluntary or involuntary exchanges that crossovers occur as a result of the inter-individual relationships among the members of a team.

In addition, Westman (2001) describes crossovers as mechanisms through which experiences, emotions, and resources are transferred within a given social and organizational context. These exchanges are also factors that contribute to the establishment of a positive work climate (e.g., a climate of psychological safety) within the team, based on increased levels of cooperation and cohesion, in a context of strong interdependence among team members (Dutton and Heaphy, 2003). In this vein, many empirical studies posit social support as a key resource (Chênevert et al., 2019) that fosters positive spirals of resource development (Meneghel et al., 2016) and promotes the achievement of individual, collective, and organizational goals (Hobfoll, 2002; Chen et al., 2015). Consistent with the conceptualization of resource caravans (Hobfoll, 2011b), we believe that the presence of social support in the work collective constitutes a central resource. Not only does this process favor the exchange and maintenance of existing resources, but it is also at the origin of the development of a pool of collective resources that will protect the team members in threatening situations. Consequently, this creates a form of collective immunity: team resilience.

Thus, we assume that team resilience is an emergent construct that arises both from the degree of resilience of each team member and from their capacity to diffuse this resource through social exchanges. This enables team members to reach a state of collective resilience, which, in turn, will nurture the potential for team resilience. As suggested by the multilevel empirical analysis by Salanova et al. (2012), employee resilience is an important and active contributor of team resilience, and this notably trough a supportive team climate. While comparing overcoming adversity with climbing, a resilient worker in the team acting as the first on the rope by providing relational resources, social support and guidance to his colleagues.

In accordance with these assumptions, we state the following:

*Proposition 2*: The worker’s individual resilience is a resource that the individual provides to their work group. It acts as a facilitator for the emergence of this construct at higher levels. In this way, it is a necessary but not sufficient condition for the emergence of team resilience.

*Proposition 3*: The level of social support among team members or within the work group moderates the emergence of collective resilience. Social support allows for the creation of bridges among individuals, which promote both the exchange and the transfer of resources. Thus, the greater the social support, the higher the intensity and speed of team resilience emergence.

Moreover, as Gucciardi et al. (2018) suggest, this collective emergent process also arises from the activation of resources due to the encounter of adversity, the key antecedent of resilience. This threatening situation triggers the collective adaptative process, and resilient team members become active contributors to the situational analysis trough mindfulness (Weick et al., 1999; Weick and Sutcliffe, 2001; Linnenluecke, 2015). Communication and informational activities are then deployed which favor the creation of shared mental models of teams’ members, what Weick and Roberts (1993) qualify as the appearance of a collective mind. In turn, this shared vision of the adversarial event enhances both coordination and sensemaking (Weick, 1993; Barton and Sutcliffe, 2023) through the activation of relational structures and resources (Powley, 2009; Linnenluecke, 2015). These will then act as bridges among team members which will facilitate the exchanges of relevant resources considering the nature of the adverse event they are confronted to (Gucciardi et al., 2018).

#### 3.2.4. Organizational resilience emergence

We conceptualize organizational resilience as a collective resource shared by the various members of the organization (Sutcliffe and Vogus, 2003; Vogus and Sutcliffe, 2007; Barasa et al., 2018). This emergent resource represents the highest level of analysis of this meta-construct and is based on the relationships or social processes that occur within the organization. While team resilience, like its emergence, is characterized by a high level of interdependence among group members (Stoverink et al., 2020), organizational resilience is based on a lower level of interdependence among workers. This relates to the frequency of interactions to carry out missions within a team, which necessitates a greater number of exchanges.

Modern organizational structures, as well as their numerous deployments of temporary cross-functional teams (e.g., project teams, committees), can be vectors of interdependence among workers, and this beyond the established framework of their core team. However, the complexity of current organizational systems may impact the interdependence of stakeholders. Indeed, the various divisions into teams, departments, and production sites are all factors that potentially reduce communicational and interactional bridges. This in turn impede the emergence of organizational resilience, as it is precisely these interactions that first enhance social support and then lead to the emergence of this third-level construct. Although these complex structures slow down emergence, they do not completely hinder it.

Furthermore, the collective and relational processes that we described as active contributors in the emergence of team resilience also apply here as the creation of a collective mind is a phenomenon enacted by organizational actors at all levels. We suggest the existence of some degree of isomorphism between the emergence of team resilience and organizational resilience. Accordingly, the underlying mechanisms we described earlier (e.g., social support, contagion, imitation, crossover) also apply here, but the fundamental differences between these two levels relates to the temporality within which their emergence occurs (Khan et al., 2019). Indeed, we assume the presence of a positive relationship between the level of interdependence among the individuals of a work collective and the speed at which the construct emerges at higher levels (i.e., team, organizational). From a temporal perspective, this implies that team resilience emerges faster than organizational resilience.

This idea is reflected in the following proposition:

*Proposition 4*: The degree of interdependence among organizational actors moderates the emergence of resilience from the individual level to the collective (i.e., team, organizational) levels, meaning that the higher the interdependence among workers, the faster higher-level resilience emerges.

If organizational resilience can emerge through the prism of the resilience of collective structures (i.e., team), then it can also emerge through additional channels. Thus, we assume that individuals’ resilience also has a direct and positive effect on the genesis and the maintenance of organizational resilience. Indeed, the positive attitudes and behaviors of a resilient worker (e.g., commitment, mobilization, performance) are levers that act at the highest levels of the organization. This perspective is consistent with many conceptualizations of bottom-up organizational constructs (e.g., organizational performance, organizational culture) (Hatch, 1993; Avey et al., 2011). This leads us to making the following two propositions:

*Proposition 5*: The worker’s individual resilience is a resource that the individual provides to their organization. It acts as a facilitator for the emergence of resilience at higher levels. In this respect, it is a necessary but not sufficient condition for the emergence of organizational resilience.

*Proposition 6*: Team resilience is a shared resource that the team provides to its organization. It acts as a facilitator for the emergence of resilience at higher levels. In this respect, it is a necessary but not sufficient condition for the emergence of organizational resilience.

**Figure 1:**
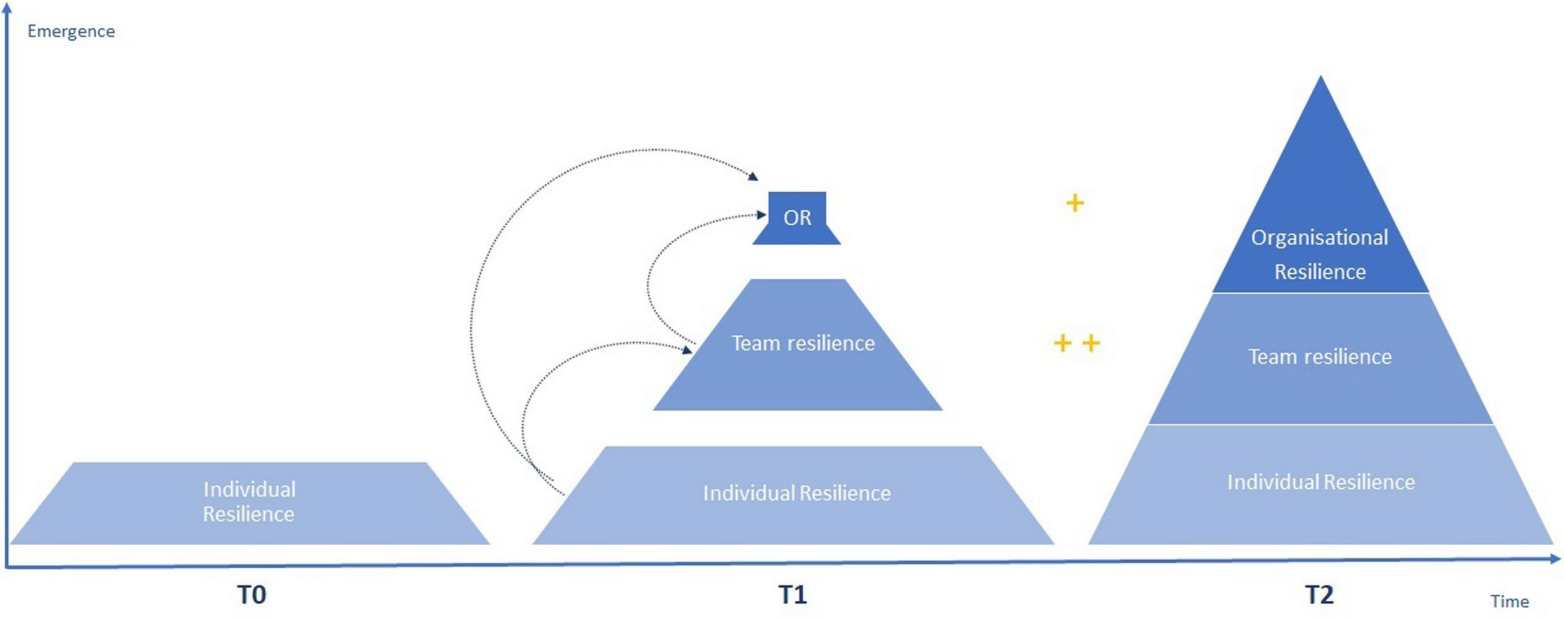
Synthesis of the bottom-up emergence process of resilience in an organizational context. *OR, Organizational Resilience; +/++, Level of interdependency between actors.

The proposals related to the emergence of resilience at work from a bottom-up perspective are summarized in Figure 1, which highlights the processes at play among the different levels of analysis of this metaconstruct. This figure also emphasizes two key elements in the mechanisms that contribute to the genesis of resilience: the level of interdependence (Stoverink et al., 2020) among stakeholders and the temporal constituent (Hobfoll et al., 2018) of these emergent processes.

In this section, we first conceptualized the emergence of organizational resilience as a bottom-up process rooted in the individual level. Yet, the resilience of organizational systems must also be studied *via* the complementary prism of top–down phenomena (Sutcliffe and Vogus, 2003; Vogus and Sutcliffe, 2007; Britt et al., 2016). Indeed, this complementary perspective allows us to highlight the complexity of this phenomenon by integrating the various ways in which organizations can foster the emergence and the maintenance of resilience at work, specifically through the implementation of appropriate formal and informal organizational practices (Chen et al., 2015).

## 4. Organizational contributions in top–down dynamics

From a theoretical perspective, Kozlowski and Klein (2000) describe top–down processes as relying on two distinct perspectives: direct and indirect effects. The former relates to the direct effects of high-level structures on lower-level units. Workplace resilience is well aligned with these statements, as we have emphasized that individual resilience, like team resilience, is influenced by a set of organizational characteristics or practices (Vera et al., 2017; Hartmann et al., 2020a,b; Raetze et al., 2021). The latter refers to the presence of indirect effects that influence the resilience of both individuals and work collectives. For example, by providing a formal and structural framework that circumscribes and regulates interactions among members of a given team, organizations impact work dynamics, which in turn influence the resilience of the team (Hartmann et al., 2020a,b).

Various authors (e.g., Sutcliffe and Vogus, 2003; Lengnick-Hall et al., 2011; Hobfoll, 2011a; Salanova et al., 2012; Akgün and Keskin, 2014; Chen et al., 2015; Hobfoll et al., 2018) emphasize the active and proactive roles that organizations play in the cultural and structural coordination of the actions that are implemented by the members of these complex sociotechnical systems (Hobfoll et al., 2018). Regarding organizational resilience, Vogus and Sutcliffe (2007) suggest that it is both a matter of identifying and disseminating the ways by which the organization has successfully overcome past crises as well as a matter of developing the pool of organizational resources to foster positive adjustments to future crises. This proactive aspect of resilience (i.e., organizational resilience capacity) is defined as “a firm’s ability to effectively absorb, develop situation-specific responses to, and ultimately, engage in transformative activities to capitalize on disruptive surprises that potentially threaten organization survival” (Lengnick-Hall et al., 2011). It can notably be achieved through the implementation of policies and practices that concern both transversal organizational aspects and human resources management (e.g., organizational communication, training, coaching, support or discussion groups) (Salanova et al., 2012; Akgün and Keskin, 2014; Vakilzadeh and Haase, 2021).

From this perspective strategic human resource management emphasizes the role of a set of HR practices, often referred to as high involvement work practices, in influencing employee behaviors and organizational outcomes. These practices are intended to increase employee participation in organizational decision-making processes (Wood et al., 2012). As emphasized by Salanova et al. (2012), with the notion of “healthy organizational resources and practices” these top–down mechanisms nurture both task resources and interpersonal resources for workers. They define these two types of job resources as follow: “Task resources are the closest to employees’ work activity, as they are related to the characteristics of the tasks themselves (task clarity, autonomy, feedback), which encourage the employee in connection with the work done, and feelings of pride and enjoyment emerge. Interpersonal resources refer to the people who employees work with and for, such as coworkers, supervisors, and customers and increase the connections employees have with the people they work for and with” (p. 790).

The theoretical approaches frequently used to explain how these HR practices improve employee well-being also refer to the demand-resource model (Bartram et al., 2012). This model states that employees are able to cope with the demands of their work and reduce negative health effects when they have a higher level of autonomy and control over their jobs. This model also stipulates that organizational demands influence the tension experienced by employees, whereas organizational resources protect employees from these tensions and generate commitment. Thus, an appropriate balance between these job demands and resources improves both the resilient capacity and the well-being of employees (Sun and Pan, 2008).

In addition, the evolution of organizational structures, toward more agility, aims not only at countering the information asymmetry in organizations, but also at minimizing the problems raised by the bounded rationality of actors (Lee and Edmondson, 2017; Vakilzadeh and Haase, 2021). Therefore, being an agile organization inevitably means to break the specialization within the companies, either by developing workstations with a wider span of control, or by promoting more horizontal communication and more autonomy, while ensuring the coordination of everyone’s actions.

In fact, the proactivity of organizations refers to an application of the concept of passageways (Hobfoll, 2011b) that fosters the emergence of resilience as well as its diffusion across the three organizational levels, through targeted organizational practices. With regard to the principles described by the COR theory (Hobfoll, 1989, 2001), we observe that by making resources available to the employees and the work collectives, the organization promotes the maintenance of a positive pool of resources (Bardoel and Drago, 2021). This protects from the risks of potential resource loss a reference to the first corollary of the COR theory: “Those with greater resources are less vulnerable to resource loss and more capable of resource gain” (Hobfoll et al., 2018, p. 106). Additionally, it leads to the potential gain of new resources for the employees, a concept also referred to as gain spirals (Hobfoll, 1989).

### 4.1. Virtuous organizational practices

By integrating complementary dimensions, all aligned with the organizational issues we described, virtuous organizational practices (Aubouin-Bonnaventure et al., 2021) constitute an important construct to consider while studying the top–down positive —direct and indirect— effects of organizational settings on resilience at work. By the term ‘virtuous organizational practices’ (Aubouin-Bonnaventure et al., 2021), these authors refer to a set of measures or values shared by the organization, such as the implementation of an organizational culture based on the support of actors at all levels (Chen et al., 2015; Hobfoll et al., 2018), the deployment of active and positive communication to disseminate key messages within the organization, and even managerial practices aligned with a participative approach. Overall, these virtuous practices promote the recognition of the work accomplished as well as social support among all organizational actors. Indeed, achieving the deployment of this organizational virtuous circle is a matter of mobilizing both individual and collective resources to create levers that will foster the emergence of another key resource, namely social support (Chen et al., 2015; Hobfoll et al., 2018).

Hobfoll and Stephens (1990) define social support as “the interactive process between individuals and their environments for the purpose of attaining behavioral or emotional assistance and is considered as one aspect of the repertoire of resources that individuals utilize to cope with stress” (p. 97). Ultimately, as highlighted by Hobfoll (1998), this construct is an indispensable component of stress management — referring to the deployment of a positive adaptation in the face of daily negative situations — but also a critical one when considering the resilience process — referring to facing occasional adverse situations.

Along these lines, virtuous organizational practices as well as the quality of interpersonal relationships at work are vectors of sensemaking and commitment as well as belonging, as they promote exchanges among the members of the organizational system (i.e., crossover) (Hobfoll et al., 2018; Barton and Sutcliffe, 2023). These exchanges then favor the creation of pools of structural and psychological resources that the workers will then be able to seize, mobilize, disseminate, and exchange. This is what Chen et al. (2015) describe as the commerce of resources: a place of symbolic exchange that allows the emergence and diffusion of collective resilience (i.e., teams, work groups, organization).

These virtuous organizational practices also nurture all three dimensions of the organizational resilience capacities, described by Lengnick-Hall et al. (2011) as: the cognitive resilience, the behavioral resilience, and the contextual resilience. Cognitive resilience captures the presence of orientation through a strong sense of purpose, vision, or values that orient employees during times of crisis. As for the behavioral resilience, it represents learned resourcefulness, counterintuitive agility, useful habits tied to organizational values, and behavioral preparedness, all of which contribute to an organization’s ability to respond to unprecedented challenges and capitalize on emerging opportunities creatively and effectively. Finally, the contextual resilience capability captures the extent to which employees feel safe (i.e., psychological safety) to take risk and developing interpersonal relationships.

In light of these various observations, we offer the following proposition:

*Proposition 7*: The implementation of virtuous organizational practices embodies passageways that promote the crossover of resources among organizational actors, leading to the emergence, maintenance, and development of resilience at work at all three levels of the organization (i.e., individual, team, organizational).

### 4.2. Temporal perspectives

In addition, following a dynamic approach of the meta-construct of workplace resilience, we suggest that the two processes involved, namely emergence and top–down dynamics, share a common developmental principle over time (Hobfoll, 1998; Hobfoll et al., 2018; Stoverink et al., 2020). It is consistent with our first proposition which conceptualizes resilience at work as a developmental construct. Indeed, time acts as a lever in the emergence of each of the three levels of resilience at work as it is associated with an accumulation of both individual and shared occupational experiences. This occurs through the overcoming of adverse situations by workers and teams over the course of days, months and years, in turn leading to resources capitalization. This implies the concurrent existence of two mutually reinforcing positive dynamics through time.

**Figure 2:**
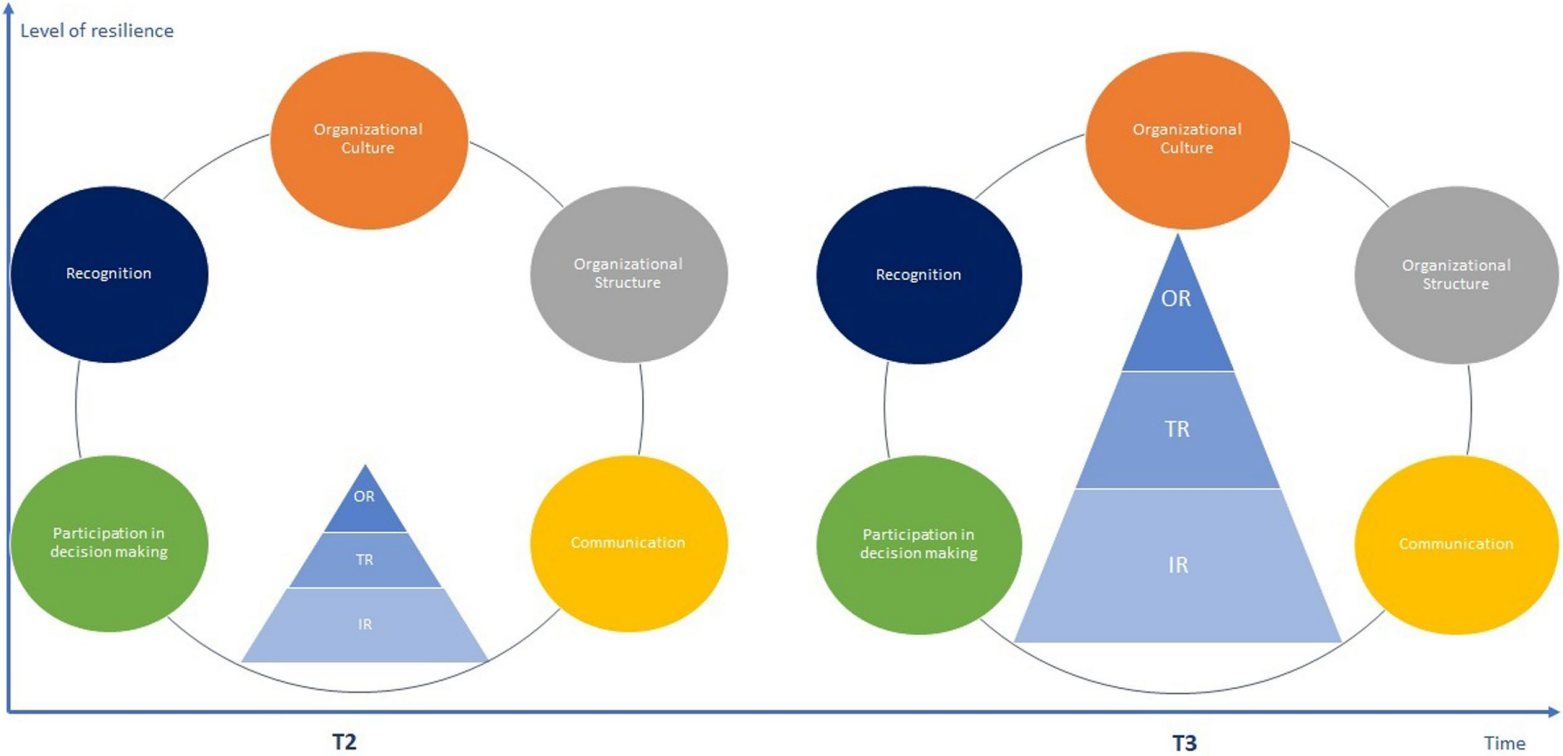
Synthesis of the top–down process. Effect of trickle-down on the development of resilience at work over time. *OR, Organizational Resilience; TR, Team Resilience; IR, Individual Resilience.

Therefore, if Figure 1 highlights the incremental emergence and growth of resilience based on its three levels over time, Figure 2, on the other hand, emphasizes the importance of continuous organizational contributions in fostering resilience at work at all its three levels, and this from a top–down perspective. Yet, these two dynamics do not unfold in a strictly sequential manner. In fact, they share a temporal stumbling block — T2 in both figures — which emphasizes the moment when the emergence of resilience at the highest levels of the organization will enable the implementation of positive spillovers mechanisms. Consequently, this crossing point marks the beginning of a virtuous organizational circle based on the simultaneous deployment of these two organizational dynamics, which draws on both the positive impact of resilience at work, and a continuous flow of emergence. Beyond the emergence dynamics presented in Figure 1, 2 emphasizes the potential for growth in the meta construct of resilience at work over time. Based on this we formulate this last theoretical proposition:

*Proposition 8*: The continuous and repeated establishment of a dynamic of resource flow, through the implementation of virtuous organizational practices, promotes the development of the meta-construct of resilience at work over time. This also results in the development of each level of resilience (i.e., individual, team/collective, organizational).

## 5. Discussion and implications

### 5.1. Theoretical implications

Our conceptual model of resilience at work theorized as a meta-construct offers a new and integrative view of this key construct for organizations, and this at all the existing levels of the organization (i.e., individual, team, organizational). This is an important contribution since, to our knowledge, there is currently no integrative model that explains the mechanisms linking resilience across the three levels of the organization, as stated by Linnenluecke (2015) when she stated: “However, there are currently few insights into how these different levels of analysis are linked to each other and how resilience can potentially be ‘scaled up’ (p. 25). Furthermore, this answers recent calls from several experts in this field of study (e.g., Sutcliffe and Vogus, 2003; Vogus and Sutcliffe, 2007; Linnenluecke, 2015; Duchek, 2020; Hartmann et al., 2020a,b; Raetze et al., 2022). Also, our conceptual proposition describes the dynamic phenomena at stake, which have not been conceptualized previously. This is achieved through the identification of three fundamental processes at play: the emergence of resilience from the individual level, the trickle down of resilience from the organizational level, and the embedding of these two in a temporal perspective, which is essential to consider in the study of this construct that we define as a dynamic process. Thus, it is an important contribution, as previous conceptual models have been focused on modeling the relationship between resilience and its determinants and consequences, without ever opening the “black box” and studying the processes intrinsically related to this construct.

Although the academic literature on workplace resilience is currently scattered, we believe that our conceptual model can be used as a foundation on which to build future theoretical and empirical research. Indeed, as described by Sutcliffe and Vogus (2003), workplace resilience is not a recent topic, but the fragmentation of both its theoretical and empirical perspectives has considerably slowed down its conceptual development, making it unclear and sometimes even worthless in both academic and professional circles. We hope that our theorization will contribute to a better understanding of this concept, which in turn will allow for a change in both its perception and mobilization. This should be reflected in a willingness to gather and build common knowledge about this topic and to build a strong conceptualization that is shared by most researchers.

Importantly, our theoretical development and its 8 propositions contribute to the resilience literature by building bridges and engaging an academic dialog between parallel and often disconnected literature. Indeed, it integrates the organizational resilience literature, usually interested in resilience capacity (e.g., Highly Reliable Organizations – HRO, Healthy and Resilient Organization – HERO model), the team resilience literature, focusing mainly on team characteristics and dynamics and finally (e.g., Gucciardi et al., 2018), the individual resilience literature whose general approach is on identifying key individual characteristics contributing to the genesis of resilience (e.g., PsyCap training). Accordingly, we break silos across both theories and levels of resilience by providing our integrative and dynamic conceptual model. By doing so, we stress the necessity of placing people and work collectives at the heart of the study of resilience at work.

In addition, we contribute to the COR theory (Hobfoll, 1989, 2001), as well as its more recent developments (Chen et al., 2015; Hobfoll et al., 2018), by positing resilience as a resource. This brings a new theoretical lens, because in these researches resilience was posited as an individual consequence of the adaptative process. Furthermore, our theory suggests that resources can be of individual or collective nature which constitutes an important consideration. Undeniably, organizations and their actors need to support each other and mutualize their resources if they aim to both maintain their functioning and provide a positive work environment.

With regards to the metatheory of resilience and resiliency provided by Richardson (2002), considered as an impactful building block in the development of the study of this concept, our theorization as various implications. First, it is aligned with the developmental perspective of the resiliency process, by suggesting that resilience at work has a growth potential over the course of occupational life and experiences. Second, if Richardson’s (2002) contribution provides a general metatheory “that crosses academic and professional boundaries” (p. 319) our theorization focuses on one specific life area and offers and in-depth explanation of the processes at play. Further, we provide a dynamic consideration of the important role of social relationships and support in the emergence and maintenance of resilience at work, whereas Richardson offers an integrated approach building on an individual perspective. Finally, this workplace specific theorization addresses important occupational issues and offers avenues for targeted interventions at all organizational levels, while Richardson’s (2002) contributions provide guidance well suited for clinical care which is more patient oriented. Accordingly, these two theories appear to be complementary by targeting two important area of life, that is the personal life and the work life.

Moreover, the scope of our theory is currently limited to the conceptualization of resilience in the professional context. However, there is a growing body of academic work that emphasizes the importance of considering issues related to the balancing and even the enrichment of different domains of life (e.g., personal, professional; Greenhaus and Powell, 2006). Consequently, as pointed out by Hobfoll et al. (2018), it will be important to examine how the personal sphere — specifically, individual resilience outside of work — contributes to the emergence of individual resilience at work. Indeed, according to the COR theoretical perspectives, the notion of a resource caravan does not have clear and defined boundaries among life domains. On the contrary, it suggests that resources, as well as the resource pools available to individuals and collectives, can overlap to enrich the different spheres of life in which individuals operate (e.g., family, work team, organization).

### 5.2. Empirical implications

We believe that the second step in this conceptual journey will consist of subjecting our theory to empirical testing. However, while it appears that there are numerous existing metrics used to measure workplace resilience (i.e., individual, team, organizational), they do not provide a suitable measure for making an appropriate assessment of our meta-construct. Thus, like other researchers (e.g., Duchek, 2020; Hartmann et al., 2020a,b; Raetze et al., 2022), we emphasize the importance of creating a new and appropriate scale of measurement that would reconstitute resilience at work as a meta-construct composed by its three sub-dimensions (i.e., individual, team, organizational). Moreover, future empirical studies will also have to consider the dynamic and temporal aspects at stake in the operationalization of our construct.

We mentioned earlier that the identification of the antecedents and consequences of resilience at work is outside the scope of our theoretical contribution. Nevertheless, it seems important to note that one logical continuation of this work could be the identification of shared, versus exclusive, antecedents at each of the three levels of resilience. From a practical perspective, this would enable the identification of the priority levers to be acted upon in an organizational setting, as a means of maximizing their positive impacts. This might involve conducting a rigorous meta-analysis to help with identifying the weighting of various antecedents (Sutcliffe and Vogus, 2003; Vogus and Sutcliffe, 2007). Unfortunately, the great disparity among the used measurement scales currently makes this work complex; hence, it is also in this respect that we stress, once again, the importance of producing a reliable and shared measurement tool.

Also, conceptualizing resilience at work as a developmental construct will require longitudinal empirical studies (Britt et al., 2016), as the widespread cross-sectional studies in our field do not allow us to highlight the emergence of resilience at work. This should be done through a rigorous analysis of the evolution of the construct over time, concerning, for example, the emergence or the development of each level over time. This will involve studying the degree of stability of each of the resilience levels to understand whether second-order constructs (i.e., team, organizational) exhibit greater degrees of constancy.

### 5.3. Practical implications

Regarding the applications of our theoretical model in workplaces, we would first like to emphasize that each workplace has specificities that make it unique. Thus, the nature of virtuous organizational practices, as well as their importance, highly depends on the professional environments under scrutiny. Indeed, in certain work environments, such as health organizations and emergency services, resilience is more prevalent, both because of the nature of the work and because of the challenges workers face on a daily basis. We hope that the proposed model will raise awareness regarding the critical issues associated with providing the appropriate individual and collective resources within organizational systems. This could result in a comprehensive and coherent strategy across and within each level of resilience, through the implementation of training on mindfulness, the establishment of spaces for discussion and group exchanges, and the promotion of a culture of support and kindness, all of which could consequently allow for the development of interdependence and social support among workers.

Finally, it seems important to emphasize that organizational systems are not isolated silos. As we have just mentioned, they are dependent on the non-work components of their stakeholders, and they are also subject to an external environment that significantly affects access to different resources (e.g., economic, social, legal constraints). Yet, both the industries and the societies in which organizations operate play key roles in the ability of organizations to adjust positively in the face of a crisis. Even if this point is outside the scope of our theory at the moment, it would seem important to consider how the external environment of the organization can promote, or, on the contrary, limit, the resilience potential of organizational systems.

### 5.4. Limits and perspectives

Throughout this paper, we have based our argument on a positive conceptualization of resilience. Indeed, the positive anchors of resilience at work (Luthar et al., 2000) have important implications for the way researchers position this construct as a key lever in the improvement of workplaces (Britt et al., 2016). Still, many scholars in our field now emphasize the importance of also considering the negative sides, or “dark sides,” of concepts that were initially studied through a positive prism, such as leadership or friendship at work (Harms et al., 2011; Pillemer and Rothbard, 2018). Regarding resilience at work, Britt et al. (2016) emphasize the necessity of not falling into the trap of conceptualizing resilience at work as an individual “responsibility,” which could lead to certain deleterious consequences in workplaces. This is what Hobfoll et al. (2018) identify as the risk of tilting toward stigmatizing non-resilient workers (i.e., victim shaming; Adler et al., 2011; Adler, 2013). It is a matter of collectively building both individual and collective resources and disseminating them within the organization. Therefore, it is not a matter of blaming a worker in difficulty because of adversity, but rather of mobilizing work groups to develop mutual support.

Finally, we would like to emphasize the importance of the awareness of organizational responsibility in the genesis and development of resilience at work. Indeed, despite the many challenges facing organizational systems, it is the responsibility of each organization to promote the establishment of a healthy and supportive work environment, particularly through the implementation of committed and meaningful organizational practices.

## Data availability statement

The original contributions presented in the study are included in the article/Supplementary material, further inquiries can be directed to the corresponding author.

## Author contributions

AG: substantial contributions to the conception and design of the work and realized multiple revisions relating to important intellectual content. DC: provided multiple revisions relating to important intellectual content. EF: provided multiple revisions relating to important intellectual content. PG: contributed to the ideation process and some of the revisions of the intellectual content. All authors contributed to the article and approved the submitted version.

## Funding

This research article benefited from the financial support of the Social Sciences and Humanities Research Council (SSHRC). Grant Number: 435-2020-1370.

## Conflict of interest

The authors declare that the research was conducted in the absence of any commercial or financial relationships that could be construed as a potential conflict of interest.

## Publisher’s note

All claims expressed in this article are solely those of the authors and do not necessarily represent those of their affiliated organizations, or those of the publisher, the editors and the reviewers. Any product that may be evaluated in this article, or claim that may be made by its manufacturer, is not guaranteed or endorsed by the publisher.

## Footnotes

^1^ https://www.merriam-webster.com/dictionary/adversity
